# A theoretical prediction of super high-performance thermoelectric materials based on MoS_2_/WS_2_ hybrid nanoribbons

**DOI:** 10.1038/srep21639

**Published:** 2016-02-17

**Authors:** Zhongwei Zhang, Yuee Xie, Qing Peng, Yuanping Chen

**Affiliations:** 1 School of Physics and Optoelectronics, Xiangtan University, Xiangtan 411105, Hunan, P.R. China; 2 Department of Mechanical, Aerospace and Nuclear Engineering, Rensselaer Polytechnic Institute, Troy, NY, 12180, USA

## Abstract

Modern society is hungry for electrical power. To improve the efficiency of energy harvesting from heat, extensive efforts seek high-performance thermoelectric materials that possess large differences between electronic and thermal conductance. Here we report a super high-performance material of consisting of MoS_2_/WS_2_ hybrid nanoribbons discovered from a theoretical investigation using nonequilibrium Green’s function methods combined with first-principles calculations and molecular dynamics simulations. The hybrid nanoribbons show higher efficiency of energy conversion than the MoS_2_ and WS_2_ nanoribbons due to the fact that the MoS_2_/WS_2_ interface reduces lattice thermal conductivity more than the electron transport. By tuning the number of the MoS_2_/WS_2_ interfaces, a figure of merit *ZT* as high as 5.5 is achieved at a temperature of 600 K. Our results imply that the MoS_2_/WS_2_ hybrid nanoribbons have promising applications in thermal energy harvesting.

Environmental pollution and energy shortages are two big concerns in modern society. Thermoelectric materials, which can convert waste heat in the environment to electricity, are expected to be helpful in resolving these two issues^1^,^2^,^3^,^4^. The energy conversion efficiencies of thermoelectric materials are measured by the so-called figure of merit *ZT* which is defined as 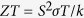, where *S* is the Seebeck coefficient, *σ* is the electronic conductance, and 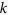 (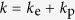) is the total thermal conductance including contributions of electrons (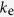) and phonons (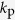). Therefore, a high-performance thermoelectric material should have high electron conductance and low thermal conductance, i.e., electron crystals and thermal glasses. However, the *ZT* values of most bulk materials are very small (much less than 1.0) because their electronic and thermal properties always have the same trends^5^,^6^,^7^,^8^.

There are extensive studies to search for high *ZT* materials. It is reported that the *ZT* values of some materials are improved significantly after nanocrystallization due to drastic changes of electronic and thermal properties^9^,^10^,^11^,^12^,^13^,^14^,^15^,^16^. For example, theoretical calculations proved that the *ZT* values of quasi-one nanowires have a larger increase than those of bulk and two-dimensional structures^2^ nanostructured bismuth antimony telluride showed experimentally higher *ZT* values than the bulk because of a sharp reduction in 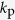
6. Beyond the intrinsic improvement, the *ZT* values of nanostructures can be further enhanced by various modifications, such as hybridization^10^,^11^,^12^, doping^13^,^14^, absorption^15^,^16^, *etc*. Previous theoretical studies indicated that hybrid nanostructures, such as SiGe alloys and hybrid BN/graphene nanoribbons, possess higher thermoelectric properties than single nanostructures^11^,^12^. Even if the thermoelectric performances of nanostructures are much better than those of bulk, most of them still cannot meet the requirements for real world applications. As such, the search for high-performance thermoelectric materials for energy harvesting applications has become a main focus in the thermoelectric field.

Recently, the thermoelectric properties of single-layer or few-layer transition metal dichalcogenides (TMD) MX_2_ (M = Mo, W, while X = S, Se *etc.*) have attracted attention^17^,^18^,^19^,^20^,^21^. MoS_2_ and WS_2_ are two typical TMDs, which are considered as excellent electronic materials because of direct band gaps and high carrier mobility. Electron transistors based on MoS_2_ and WS_2_ have been reported and show high electronic performance, while the thermal conductivities of the two nanosheets are relatively low^22^,^23^,^24^,^25^,^26^. Therefore, MoS_2_ and WS_2_ monolayer should have high *ZT* values, which have been proven by previous theoretical studies^18^,^19^,^21^. Meanwhile, some interesting MoS_2_/WS_2_ hybrid nanostructures have been synthesized experimentally and studied theoretically^27^,^28^,^29^,^30^. However, the hybrid MoS_2_/WS_2_ nanoribbons have not been synthesized yet to the best of the authors’ knowledge. Their thermoelectric properties are still unknown.

In this paper, thermoelectric transport in MoS_2_/WS_2_ hybrid nanoribbons is studied using nonequilibrium Green’s function (NEGF) methods combined with first-principles and molecular dynamics methods^31^,^32^,^33^,^34^,^35^. The hybrid nanoribbons show high-performance thermoelectric properties compared to pure MoS_2_ and WS_2_ nanoribbons. Furthermore, the *ZT* values can be enhanced by modulating the number of interfaces in the structures, which approach 5.5 at 600 K and 4.0 at 300 K, respectively. The variations of the Seebeck coefficient, electronic, and thermal conductances are analyzed to interpret the enhanced thermoelectric properties. The super high *ZT* values indicate that the MoS_2_/WS_2_ hybrid nanoribbons are ideal high-performance thermoelectric materials with high energy conversion efficiencies.

## Results and Discussion

**Figure 1:**
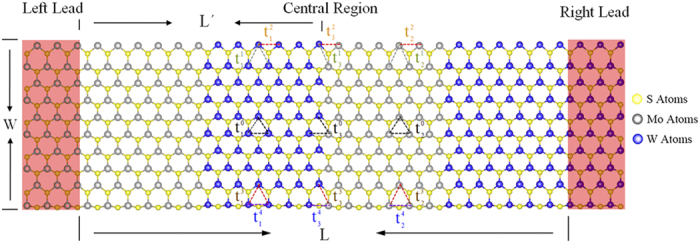
Atomic structures of zigzag-edge MoS_2_/WS_2_ hybrid nanoribbons. The model can be divided into three parts – a central scattering region and two (left and right) leads shown in the red boxes. The central scattering region, length *L* and width *W*, is a finite superlattice which consists of *N* periods. The length of each period is *L*′, and thus *N* = *L*/*L*′ (here *N* = 2). 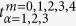 represents the hopping integrals between atoms, where the superscript 0 is set for internal atoms while others are set for edge atoms, and the subscripts 1, 2, and 3 are set for Mo-Mo, W-W, and Mo-W atoms, respectively.

The MoS_2_/WS_2_ hybrid nanostructures, as shown in Fig. 1, can be divided into three parts, one central scattering region and two (left and right) leads. The central region consists of finite periodic structures. Each period has one finite MoS_2_ nanoribbon and one finite WS_2_ nanoribbon. The two leads are semi-infinite MoS_2_ or WS_2_ nanoribbons. The length and width of the central region are labeled as *L* and *W*, respectively, and the length of a period of the central region is labeled as *L*′. Therefore, 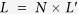, where *N* is the periodic number of the central scattering region.

A tight-binding (TB) Hamiltonian is used to describe the electronic properties of the hybrid structures,


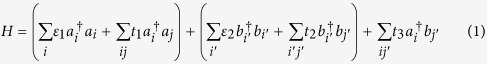


where the three terms represent the Hamiltonians of the MoS_2_ nanoribbons, WS_2_ nanoribbons, and their interactions, respectively. 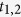 and 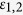 are the third-order hopping integrals for the nearest-neighbor atoms and site energies, which can be obtained from the GGA parameters in ref. 36. In ref. 36, the Hamiltonians of MoS_2_ and WS_2_ are described by three atomistic *d* orbitals of transition metal atoms, because the band edges mostly consist of 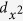, 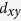, and 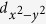 orbitals, while the hopping integral 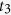 for the interaction of Mo and W atoms are taken to be the average values of 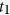 and 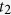, i.e., 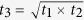. To reflect the reconstruction of the ribbon edges, the hopping integrals are varied and reset as 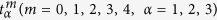, where *m* = 0 for internal atoms and 1, 2, 3, and 4 are for edged atoms, as shown in Fig. 1. The values of 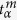 are inversely proportional to the corresponding bond lengths. According to the Hamiltonian in Eq. (1), we can use the NEGF method to calculate the electronic transport properties, including electronic conductance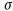, Seebeck coefficient *S*, and electronic thermal conductance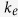. To verify the reliability of the TB parameters, we compare the electronic density of states (DOS) calculated by NEGF and density functional based tight binding (DFTB) methods^37^,^38^. The results indicate that the TB parameters can present the same energy gaps to those of the DFTB method, and the DOS profiles based on the TB parameters are also approximately similar to those of the DFTB method (see Fig. S1 in the Supplementary Information). For thermal transport properties, the phonon thermal conductance of the nanoribbons can be calculated using a harmonic approximation. One just needs to substitute the Hamiltonian matrix *H* by the mass-weighed force constant matrix *K*. Please see the Methods section for more details.

**Figure 2:**
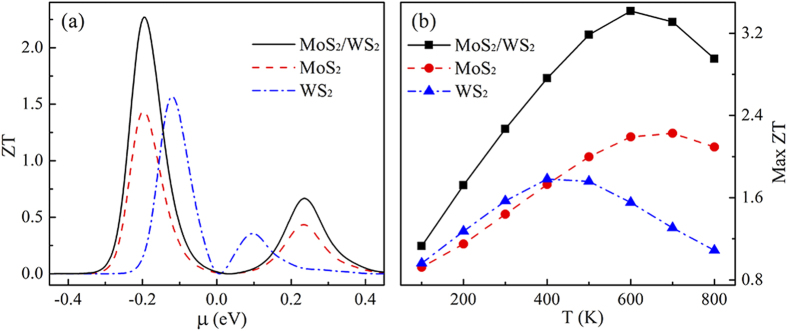
(**a**) *ZT* as a function of chemical potential 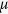 for MoS_2_/WS_2_ (*N* = 1), MoS_2_, and WS_2_ nanoribbons, respectively, at *T* = 300 K. (**b**) The Max *ZT* value as a function of temperature for MoS_2_/WS_2_ (*N* = 1), MoS_2_, and WS_2_ nanoribbons.

We calculate *ZT* as a function of chemical potential 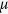 at *T* = 300 K for a MoS_2_/WS_2_ hybrid nanoribbon (*L* = 8.37 nm and *W* = 2.34 nm) where the central region only consists of one MoS_2_/WS_2_ interface, as shown in Fig. 2(a). The results for pure MoS_2_ and WS_2_ nanoribbons with the same size are also shown for comparison. It is found that the maximum values of *ZT* (Max *ZT*) for the three structures all appear at 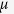 < 0, moreover, the Max *ZT* of the MoS_2_/WS_2_ nanoribbon is larger than those of the two pure structures. The Max *ZT* of the hybrid nanoribbon is 2.3, while values for WS_2_ and MoS_2_ are 1.6 and 1.5, respectively. Therefore, after hybridization, *ZT* values of the pure nanoribbons are increased approximately by 1.5 times at room temperature.

The effect of temperature *T* on the Max *ZT* of the three structures is illustrated in Fig. 2(b). All of the *ZT* curves display the same trend—an increase at low temperature to the maximum followed by reduction at high temperature. With the variation of *T*, the highest *ZT* of the MoS_2_/WS_2_ hybrid nanoribbon appears at *T* = 600 K with a value of 3.5 while the maximum values for the pure MoS_2_ and WS_2_ nanoribbons are only 2.3 (at 700 K) and 1.8 (at 400 K), respectively. Moreover, the *ZT* values of hybrid nanoribbons are higher than those of pure nanoribbons at any temperature. This further indicates that the hybridization enhances thermoelectric efficiency drastically.

**Figure 3:**
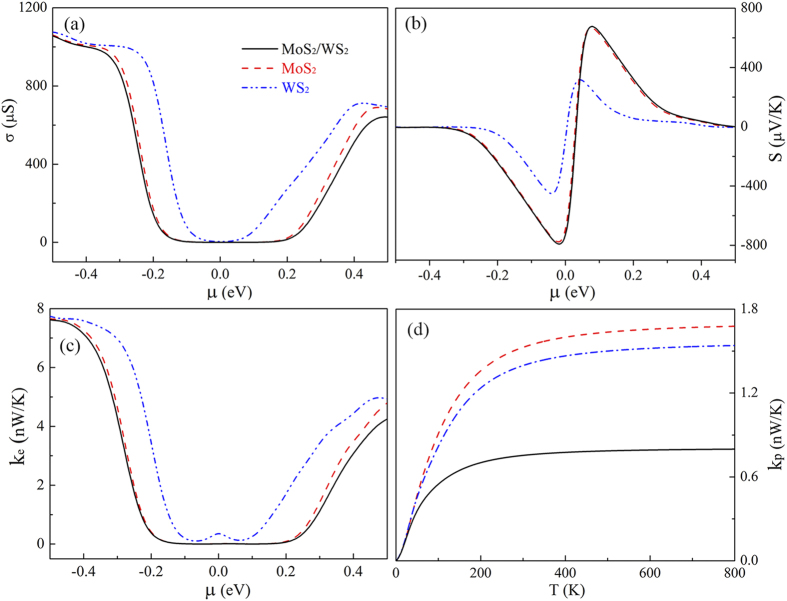
(**a**) *σ*, (**b**) *S*, and (**c**) *k*_e_ of MoS_2_/WS_2_ (*N* = 1), MoS_2_, and WS_2_ nanoribbons as a function of chemical potential 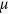, respectively, at *T* = 300 K. (**d**) *k*_p_ of MoS_2_/WS_2_ (*N* = 1), MoS_2_, and WS_2_ nanoribbons as a function of temperature *T*.

To scrutinize the mechanism of the increment of *ZT* in hybrid structures over pure ones, we calculate their electronic and thermal properties, such as *σ*, *S*, *k*_e_, and *T*_p_. Equations (2, 3, 4) demonstrate that *σ*, *S*, and *k*_e_ are functions of chemical potential 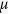 and temperature *T*, while *k*_p_ is only a function of temperature *T*. Figure 3(a–c) show *σ*, *S*, and *k*_e_ as a function of 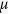 for the three structures at *T* = 300 K. Seen from Fig. 3(a), the *σ* curve of the WS_2_ nanoribbon is somewhat similar to that of the MoS_2_ nanoribbon, except the threshold values of the latter are smaller than that of the former, which is mainly because the band gap of the WS_2_ nanoribbon is smaller than that of the MoS_2_ nanoribbon. For the MoS_2_/WS_2_ hybrid nanoribbon, its energy gap should be equal to the wider one, i.e., equal to that of MoS_2_ nanoribbon. The results of DFTB calculation further confirm this point (see Fig. S1 in the Supplementary Information). Therefore, the *σ* curve of the hybrid nanoribbon has the same threshold as that of the MoS_2_ nanoribbon. Meanwhile, the interface scattering to the electrons is weak due to the similarity of the electronic properties of MoS_2_ and WS_2_, and thus *σ* values of the hybrid nanoribbon are just a little smaller than those of the pure nanoribbons. The maximum *S* usually depends linearly on the band gap^39^,^40^, therefore, one can see from Fig. 3(b) that the *S* values of hybrid and MoS_2_ nanoribbons are larger than that of WS_2_ nanoribbons. The electronic thermal conductance *k*_e_ shown in Fig. 3(c) almost coincides with the behavior of electron conductance *σ*, because both of them are attributed to the contribution of electrons. Figure 3(d) presents another *ZT* factor, the phononic thermal conductance *k*_p_ as a function of *T*. The MoS_2_ nanoribbon exhibits a higher *k*_p_ than the WS_2_ nanoribbon while the *k*_p_ of the MoS_2_/WS_2_ hybrid nanoribbon is the lowest, which is only about 0.5 times that of WS_2_ and MoS_2_ nanoribbons. From these results, one can find that the improved *ZT* value of the hybrid nanoribbon is mainly originated from the sharp decrease of phononic thermal conductance *k*_p_ while the effect of the interface on *σ*, *S*, and *k*_e_ is relatively small.

**Figure 4:**
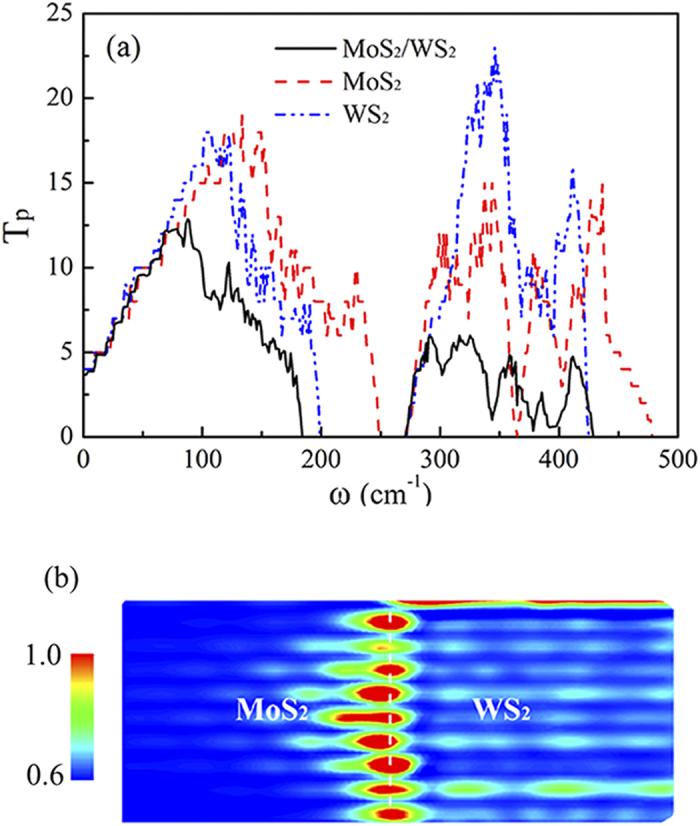
(**a**) Phonon transmission coefficient *T*_p_ of MoS_2_/WS_2_ (*N* = 1), MoS_2_, and WS_2_ nanoribbons as a function of frequency *ω*. (**b**) Phonon LDOS in the MoS_2_/WS_2_ (*N* = 1) nanoribbons at *ω = *200.0, 343.6, and 400.0 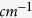. The color bar presents the strength of phonon localization.

In order to understand the drop of *k*_p_ in the hybrid structure, we compare the phonon transmissions 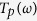 in the MoS_2_, WS_2_, and hybrid nanoribbons, as shown in Fig. 4(a). The comparison reveals that two reasons cause the drop of *k*_p_ in the hybrid structure. One is the shrinkage of the spectrum ranges where have effective phonon transmission. For example, the gaps between acoustical and optical phonons for MoS_2_ and WS_2_ nanoribbons are 24 and 73 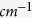, respectively, while that for hybrid nanoribbon is 87 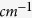. The other is the reduction of the transmission coefficients *T*_p_. One can find that, as 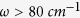, 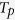 of all phonons in the hybrid structure are smaller than those in the pure nanoribbons. The two aspects demonstrate that the interface in the hybrid nanoribbon vastly weaken the phonon transport. The LDOS in the hybrid structures in Fig. 4(b) further illustrates this point. There are many localized phonons at the interface, which acts as a potential barrier blocking the phonons from left to right. It is noted that the phonon localization at the nanoribbon edges is small although the edges are unpassivated. We also compare thermal conductances of nanoribbons with different types of edges, such as unpassivated, S-half passivated^41^,^42^ and periodic edges (see Fig. S2(c) in the Supplementary Information). The results indicate that the effect of dangling bonds, phonon localization, and scattering at the edges on the thermal transport is very weak.

**Figure 5:**
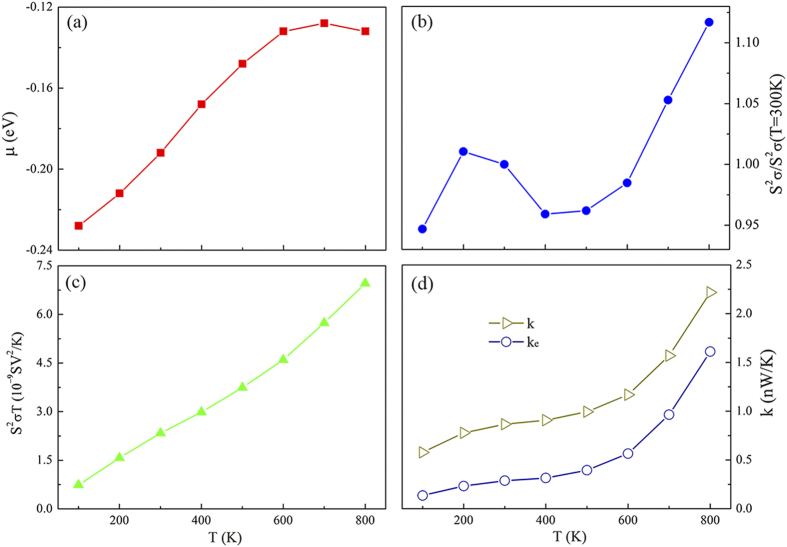
(**a**) 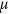, (**b**) 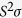, (**c**) 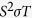 and (**d**) *k*, *k*e of MoS_2_/WS_2_ (*N* = 1) nanoribbons as a function of temperature *T*, corresponding to Max *ZT* in Fig. 2(b). The 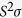 is normalized by the 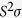 at *T* = 300 K, 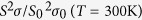.

**Figure 6:**
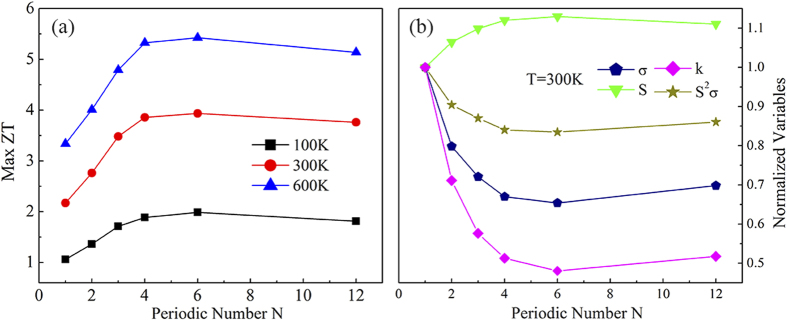
(**a**) The Max *ZT* value of the MoS_2_/WS_2_ hybrid nanoribbon as a function of periodic number *N*, at *T* = 100 K, 300 K, and 600 K. (**b**) Normalized variables *σ*, *S*, *k,* and *S*^2^*σ* as a function of periodic number *N*, at *T* = 300 K. The values are normalized by the values for *N* = 1.

To analyze the effect of temperature *T* on the Max *ZT* of the MoS_2_/WS_2_ hybrid nanoribbon, we calculate the variations of 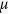, 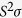, 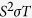, and *k* (*k*_e_) corresponding to the Max *ZT* under temperature *T*, as shown in Fig. 5. It is seen from Fig. 5(a) that the chemical potential 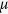 gradually shifts to the Fermi level with the increase of *T*. The variation of thermopower 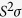 in Fig. 5(b) is irregular due to the different chemical potential 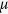 and the small amplitude of variation, indicating the effect of temperature *T* on the thermopower is weak. This is further proved by the nearly linear relation between 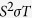 and *T* in Fig. 5(c). Therefore, the effect of temperature *T* on the Max *ZT* is determined by the relation of thermal conductance *k* and *T*. The total thermal conductance *k* is composed of electronic thermal conductance *k*_e_ and phononic thermal conductance *k*_p_. These three quantities as a function of temperature are plotted in Fig. 5(d) for comparison. The total thermal conductance *k* increases slowly with the temperature as *T* < 600 K and then increases rapidly as *T* > 600 K, which is induced by the sharp increase of *k*_e_ after *T* > 600 K. The variation of *k*_e_ with *T* is related to the shift of chemical potential 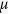 and the Fermi distribution. As a result, the highest *ZT* value appears at *T* = 600 K. Because the MoS_2_/WS_2_ interface reduces the phononic thermal conductance significantly with small penalty on other components, we can further increase the *ZT* by introducing more interfaces in the system. This could be achieved by enlarging the spatial frequencies of interfaces, which is equivalent to period number *N* while the total length is fixed. Figure 6(a) shows Max *ZT* values for MoS_2_/WS_2_ hybrid nanoribbons whose central scattering region includes *N* periods (*N* units of MoS_2_/WS_2_). The length 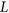 of the hybrid nanoribbons is fixed at 8.37 nm, and thus the increase in periodic number *N* represents the corresponding decrease of the periodic length 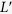. One can find that the Max *ZT* increases with *N* as *N* < 6, and then decreases as *N* further increases. The highest *ZT* exists at *N* = 6, and the values at *T* = 100, 300, and 600 K are 2.0, 4.0, and 5.5, respectively, which are 2 ~ 3 times those of pure WS_2_ and MoS_2_ nanoribbons. Obviously, the multi-periodic hybrid structures further enhance the thermoelectric properties.

To examine the effect of periodic number *N* on *ZT* as well as its components, we study the normalized *σ*, *S*, 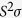, and *k* for multi-periodic hybrid nanoribbons at *T* = 300 K. They vary as a function of the periodic number *N*, as shown in Fig. 6(b). The normalized *σ* and *k* decrease as *N* increases and *N* < 4. This is because the number of the interfaces in the central scattering region increases with the periodic number *N*, which strengthens the scattering of electrons and phonons and thus *σ* and *k* decrease. As discussed above, the effect of the interfaces on the phonon transport is larger than that on the electron transport, and thus the decrease speed of *k* is faster. As *N* > 4, the central region gradually becomes a super lattice, therefore *σ* and *k* decrease slowly at first and then increase with *N*. On the other hand, the variation of *S* is inversely proportional to that of *σ* and *k*. It increases with *N* as *N* < 4 and then decreases slowly. The variation of 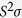 with *N* is small, indicating that the improved *ZT* values for the multi-periodic structures are still originated from the sharp decrease of *k*.

As mentioned above, in-plane lateral hybrid MoS_2_/WS_2_ heterojunctions have been synthesized by ambient-pressure chemical vapor deposition (CVD) and single-step vapor phase growth^27^,^28^. Therefore, the hybrid MoS_2_/WS_2_ nanoribbons with one interface (*N* = 1) in Fig. 1 can be obtained by cutting these two-dimensional heterojunctions^43^. Although the hybrid MoS_2_/WS_2_ heterojunctions with multiple interfaces have not been reported up to date, these heterojunctions are expected to be synthesized in the near future, by following the similar synthetic process of periodic BN/graphene heterostructures created by lithography patterning and sequential CVD growth steps^44^. Then, the hybrid MoS_2_/WS_2_ nanoribbons with periodic interfaces can also be obtained. The high-performance thermoelectric properties of these hybrid nanoribbons make them have promising applications in thermal energy harvesting.

## Summary

In summary, we study thermoelectric properties of MoS_2_/WS_2_ hybrid nanoribbons, by using the NEGF method combined with first-principles and molecular dynamics methods. The hybrid nanoribbons exhibit super high thermoelectric properties. With the drastic reduction of *k*_p_ and little change in the electronic properties, the maximum *ZT* of the hybrid nanoribbon with one interface is increased to 1.5 ~ 2 times that of pure nanoribbons. Moreover, the *ZT* values can be further increased by modulating the interface number. The highest *ZT* value of hybrid nanoribbons can approach 4.0 at 300 K and 5.5 at 600 K. Therefore, MoS_2_/WS_2_ hybrid nanoribbons are very promising materials for high-performance thermoelectric devices.

## Methods

In this section we have outlined some key steps in applying the NEGF approach for the electronic and phononic transport. We can use the NEGF method to calculate the electronic transmission coefficient 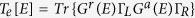
45,46, where 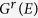 and 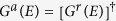 are the retarded and advanced Green’s functions which included the two leads’ effects, and 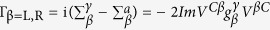 is the coupling function of the 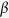 lead. Then the electronic conductance 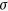, Seebeck coefficient *S,* and electronic thermal conductance 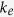 can be calculated based on the Onsager’s relations and Landauer’s theory of quantum transport:^5^,^11^,^33^


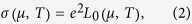



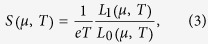



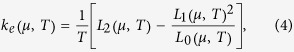


where 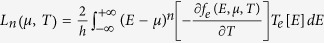 is the Lorenz integral and 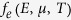 is the Fermi-Dirac distribution function at the chemical potential 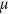 and temperature *T*.

Thermal transport properties of the hybrid nanoribbons can be calculated as follow. At first, the Stillinger-Weber (SW) potential^47^ parameters used to describe the interatomic interactions in the hybrid nanoribbons can be obtained by the software GULP based on molecular dynamics scheme^48^,^49^. These parameters can fit well the phonon dispersions calculated by the first-principles method. Then, the force constants matrices *K* of the hybrid structures can be also obtained by GULP according to the potentials. In the GULP, the force constant is given by the second derivatives with respect to the potential energy, and thus they only include the harmonic components. The detailed procedures for dealing with the potential parameters and force constants can be found in the Supporting Information. After *K* is obtained, the phonon transmission coefficient 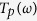 can be calculated, according to the NEGF procedure in analogy to that of calculating electronic transmission coefficient^34^,^35^,^50^,^51^. Finally, the lattice thermal conductance is given by 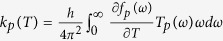, where 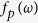 is the Bose-Einstein distribution function for heat carriers. We have therefore all the ingredients required to calculate *ZT*. Meanwhile, the LDOS at site *j*: 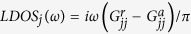 can also be calculated.

## Additional Information

**How to cite this article**: Zhang, Z. *et al.* A theoretical prediction of super high-performance thermoelectric materials based on MoS_2_/WS_2_ hybrid nanoribbons. *Sci. Rep.* **6**, 21639; doi: 10.1038/srep21639 (2016).

## Supplementary Material

Supplementary Information
