# Supplementary material for: A theoretical prediction of super high-performance thermoelectric materials based on MoS2/WS2 hybrid nanoribbons
Source: Sci Rep. 2016 Feb 17;6:21639. doi: 10.1038/srep21639 (PMC4756374; doi:10.1038/srep21639)
Supplement: Supplementary Information [file srep21639-s1.docx]

**Supplementary Information**

**A theoretical prediction of super high-performance thermoelectric materials based on MoS_2_/WS_2_ hybrid nanoribbons**

Zhongwei Zhang^1^, Yuee Xie^1†^, Qing Peng^2^ and Yuanping Chen^1*^

**S1. DOS from NEGF and DFTB calculations**

The DFTB calculations are carried out by using DFTB+ software.[^1^](#_ENREF_1)^,^[^2^](#_ENREF_2) The Slater-Koster parameters for DFTB calculations were created following the approach described by Oliveira et *al.*[^3^](#_ENREF_3) and Seifert et *al.*,[^4^](#_ENREF_4) and have been validated by band structure comparison with DFT results. The DFTB method has been widely used to investigated the electric properties of MoS_2_ and WS_2_ structures with larger number of atoms.[^5-7^](#_ENREF_5)

The DOS of NEGF approach is calculated from following formula:

$DOS=ImTr(G^{r})/\pi$ (S1)

The $G^{r}$ is the retarded Green's functions which has included the effects of left lead and right lead.


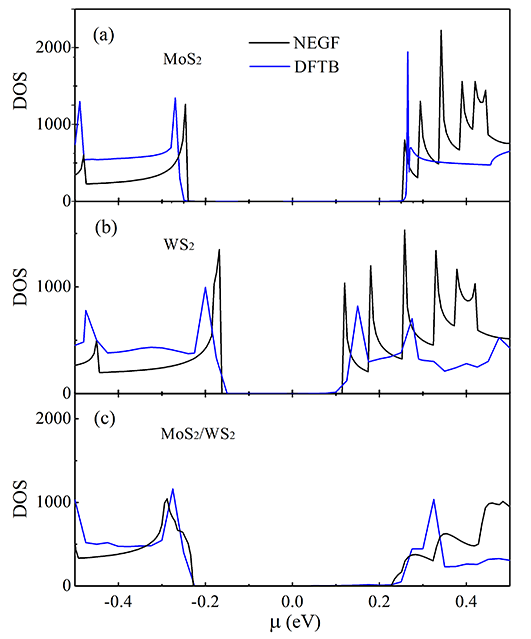


Figure. S1. Density of states (DOS) from NEGF and DFTB calculations, of (a) MoS_2_ nanoribbons, (b) WS_2_ nanoribbons and hybrid MoS_2_/WS_2_ (N=1) nanoribbons.

**S2. Thermal conductance *k*_p_ of nanoribbons with different edge condition**


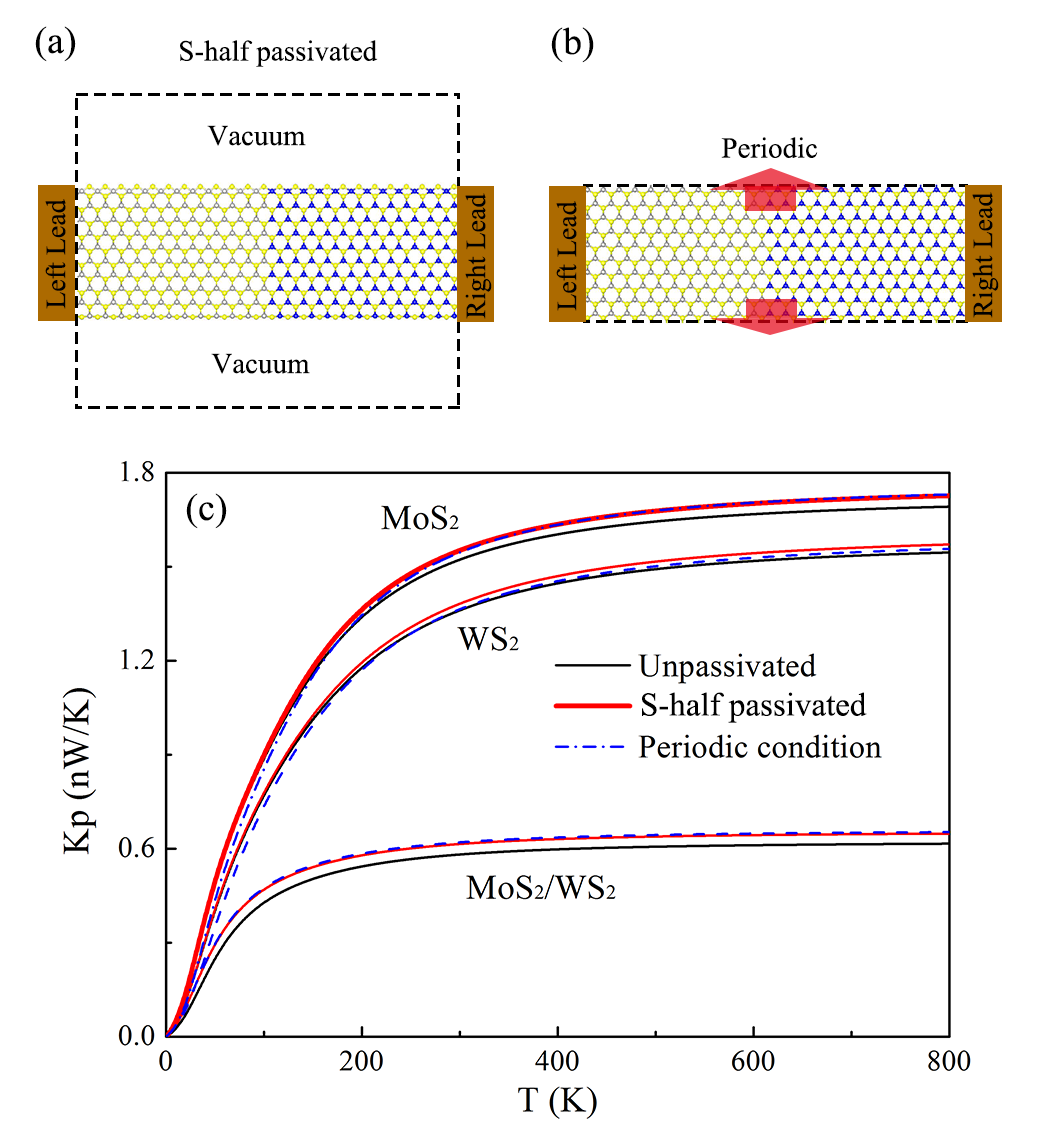


Figure S2. Atomic structure of hybrid MoS_2_/WS_2_ nanoribbons with (a) S-half passivated edge and (b) periodic condition. (c) Thermal conductance *k*_p_ of MoS_2_, WS_2_ and MoS_2_/WS_2_ hybrid nanoribbons with unpassivated edge, S-half passivated and periodic edge, respectively.

**S3. Stillinger-Weber (SW) potential parameters for MoS_2_/WS_2_ hybrid nanoribbons**

In recently a SW potential for single layer MoS_2_ (SLMoS_2_) is reported by Jiang *et al.*,[^8^](#_ENREF_8) the results show that this potential formula well describes SLMoS_2_ interactions. In consideration of the very similar structural characteristics between MoS_2_ and WS_2_,[^9^](#_ENREF_9)^,^[^10^](#_ENREF_10) we have fitted a set of SW parameters to describe the interactions in SLWS_2_. The total potential energy of system with *N* atoms from SW potential is defined as follows:[^11^](#_ENREF_11)^,^[^12^](#_ENREF_12)

$$\Phi\left( 1,\ldots,N \right)=\sum_{i<j} V_{2}\left( i,j \right)+\sum_{i<j<k} V_{3}\left( i,j,k \right). \text{(}\text{S}\text{2}\text{)}$$

The two-body interaction takes following form:

$$V_{2}=\epsilon A\left( B\sigma^{p}r_{ij}^{-p}-\sigma^{q}r_{ij}^{-q} \right)e^{[\sigma\left( r_{ij}-a\sigma\right)^{-1}]}. \text{(}\text{S}\text{3}\text{)}$$

The three-body interaction is

$$V_{3}=\epsilon\lambda e^{\left[ \gamma\sigma\left( r_{ij}-a\sigma\right)^{-1}+\gamma\sigma\left( r_{jk}-a\sigma\right)^{-1} \right]}\left( \cos\theta_{jik}-\cos\theta_{0} \right)^{2}\text{, (}\text{S}\text{4}\text{)}$$

where *θ*_0_ is the initial angle.

The code GULP[^13^](#_ENREF_13) was used to calculate the fitting parameters for the SW potential, where the SW parameters were fit to the phonon spectrum of SLWS_2_. The phonon spectrum plays a crucial role for the thermal conductivity. Moreover, the acoustic velocities from phonon spectrum are closely related to the mechanical properties of the materials. The standard phonon spectrum can be obtained from density functional theory (DFT) calculations. Such calculations were performed using Vienna *Ab-initio* Simulation Package (VASP)[^14^](#_ENREF_14) with projector augmented wave (PAW) pseudopotentials,[^15^](#_ENREF_15) and Perdew, Burke, and Ernzerhof exchange-correlation functionals.[^16^](#_ENREF_16) The structural relaxation was done for the unit cell with a 12 × 12 × 1 Monkhorst-Pack grid of k sampling. A 16.0 Å vacuum space along the c-axis was used to eliminate the interaction emerging from periodic boundary condition calculations. Then the PHONOPY code was used to calculate phonon spectrum by using the real space supercell approach.[^17^](#_ENREF_17) A 4 × 4 × 1 supercell with 3 × 3 × 1 k sampling for Brillouin zone integration was used for IFC calculation.


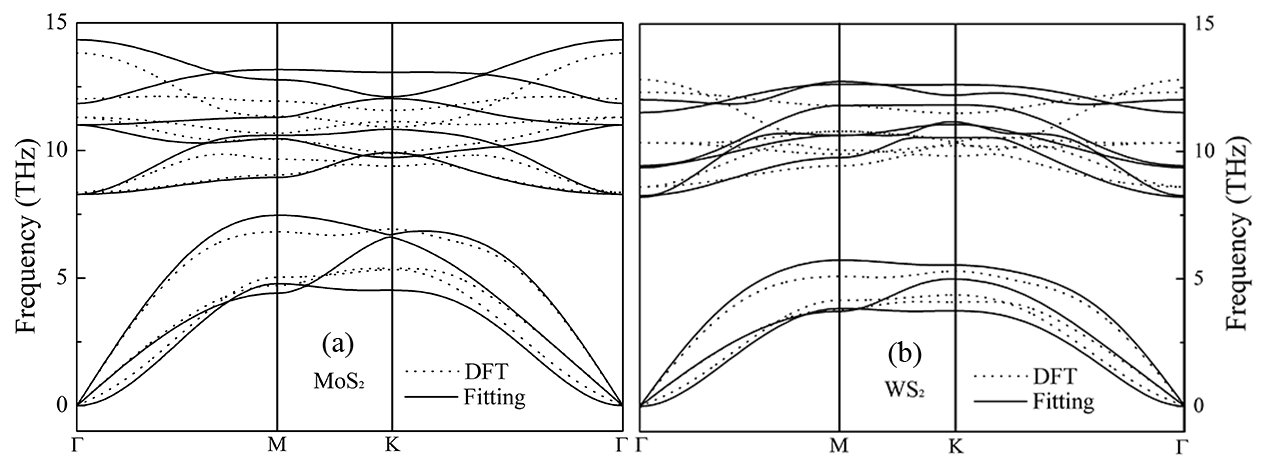


Figure. S3. Phonon spectrum for (a) SLMoS_2_ and (b) SLWS_2_, along the Г-M-K-Г directions in the Brillouin zone. The fitting results (solid line) from the fitted SW potential and DFT results (dash line) both are presented.

Figure S2(b) shows the fitting results for the phonon spectrum of SLWS_2_ along the Г-M-K-Г directions in the Brillouin zone. Moreover, the phonon spectrum for SLMoS_2_ with fitted SW parameters from Ref. [8](#_ENREF_8) TABLE I and II, which we have adapted in our simulations to calculate the force constant of MoS_2_ region, is shown in Fig. S2(a). For comparison, the phonon spectrums from DFT are also shown. As one can see, both the results from Ref [^8^](#_ENREF_8) and our fitted potential are well matched with DFT results, especially for the acoustic branch which is the primary energy carriers in thermal transport. The fitted SW parameters for SLWS_2_ which we have adapted to calculate the force constant of WS_2_ region are shown in Tables SI and SII.


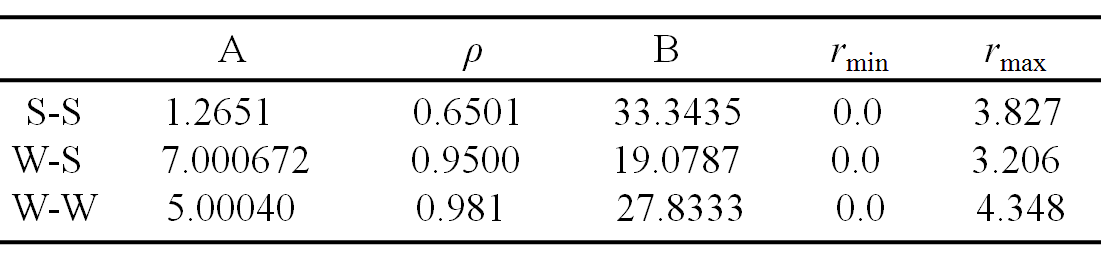


TABLE SI. The two-body (bond bending) SW potential parameters for GULP. Energy parameters are in the unit of eV. Length parameters are in the unit of Å.


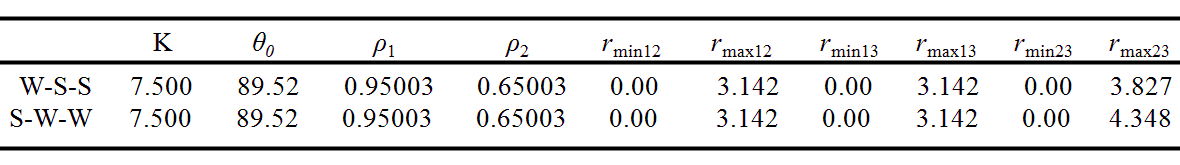


TABLE SII. The three-body (angle bending) SW potential parameters for GULP. Energy parameters are in the unit of eV. Length parameters are in the unit of Å.

Beside the well matched phonon spectrum, we also relaxed the primitive cell with the fitted SW potential in GULP. The results show that the lattice parameters is 3.107 Å, which is only 0.74% mismatch with DFT.

Based on the SW potential parameters for MoS_2_ and WS_2_ region, the parameters between MoS_2_ and WS_2_ region are taken to be the average values. In consideration of the fitted SW potential parameters for SLMoS_2_ and SLWS_2_ well describe the phonon dispersion relations and structure parameters, we think the results calculated by NEGF with SW potential is well described the phonon transport properties.

**References**

1. Aradi B., Hourahine B. & Frauenheim T. DFTB+, a Sparse Matrix-Based Implementation of the DFTB Method†. *J. Phys. Chem. A* **111**, 5678-5684 (2007).

2. Pecchia A., Penazzi G., Salvucci L. & Di Carlo A. Non-equilibrium Green's functions in density functional tight binding: method and applications. *New J. Phys.* **10**, 065022 (2008).

3. Oliveira A. F., Seifert G., Heine T. & Duarte H. A. Density-functional based tight-binding: an approximate DFT method. *J. Braz. Chem. Soci.* **20**, 1193-1205 (2009).

4. Seifert G., Porezag D. & Frauenheim T. Calculations of molecules, clusters, and solids with a simplified LCAO-DFT-LDA scheme. *Int. J. Quantum Chem.* **58**, 185-192 (1996).

5. Seifert G. *et al.* Structure and Electronic Properties of MoS2 Nanotubes. *Phys. Rev. Lett.* **85**, 146-149 (2000).

6. Ghorbani-Asl M., Borini S., Kuc A. & Heine T. Strain-dependent modulation of conductivity in single-layer transition-metal dichalcogenides. *Phys. Rev. B* **87**, (2013).

7. Erdogan E., Popov I. H., Enyashin A. N. & Seifert G. Transport properties of MoS2 nanoribbons: edge priority. *Eur. Phys. J. B* **85**, (2012).

8. Jiang J. W., Park H. S. & Rabczuk T. Molecular dynamics simulations of single-layer molybdenum disulphide (MoS2): Stillinger-Weber parametrization, mechanical properties, and thermal conductivity. *J. Appl. Phys.* **114**, 064307 (2013).

9. Ataca C., Şahin H. & Ciraci S. Stable, Single-Layer MX2Transition-Metal Oxides and Dichalcogenides in a Honeycomb-Like Structure. *J. Chem. Phys. C* **116**, 8983-8999 (2012).

10. Ramakrishna Matte H. *et al.* MoS2 and WS2 analogues of graphene. *Angew. Chem.* **122**, 4153-4156 (2010).

11. Jiménez Sandoval S., Yang D., Frindt R. F. & Irwin J. C. Raman study and lattice dynamics of single molecular layers of MoS2. *Phys. Rev. B* **44**, 3955-3962 (1991).

12. Dobardžić E., Milošević I., Dakić B. & Damnjanović M. Raman and infrared-active modes in MS2 nanotubes (M=Mo, W). *Phys. Rev. B* **74**, 033403 (2006).

13. Gale J. D. GULP: A computer program for the symmetry-adapted simulation of solids. *J. Chem. Soci., Faraday Trans.* **93**, 629-637 (1997).

14. Kresse G. & Furthmüller J. Efficient iterative schemes for \textit{ab initio} total-energy calculations using a plane-wave basis set. *Phys. Rev. B* **54**, 11169-11186 (1996).

15. Blöchl P. E. Projector augmented-wave method. *Phys. Rev. B* **50**, 17953-17979 (1994).

16. Perdew J. P., Burke K. & Ernzerhof M. Generalized Gradient Approximation Made Simple. *Phys. Rev. Lett.* **77**, 3865-3868 (1996).

17. Togo A., Oba F. & Tanaka I. First-principles calculations of the ferroelastic transition between rutile-type and CaCl2-type SiO2 at high pressures. *Phys. Rev. B* **78**, 134106 (2008).
